# Multiple alterations in glutamatergic transmission and dopamine D2 receptor splicing in induced pluripotent stem cell-derived neurons from patients with familial schizophrenia

**DOI:** 10.1038/s41398-021-01676-1

**Published:** 2021-10-25

**Authors:** Kana Yamamoto, Toshihiko Kuriu, Kensuke Matsumura, Kazuki Nagayasu, Yoshinori Tsurusaki, Noriko Miyake, Hidenaga Yamamori, Yuka Yasuda, Michiko Fujimoto, Mikiya Fujiwara, Masayuki Baba, Kohei Kitagawa, Tomoya Takemoto, Nanaka Gotoda-Nishimura, Tomohiro Takada, Kaoru Seiriki, Atsuko Hayata-Takano, Atsushi Kasai, Yukio Ago, Satoshi Kida, Kazuhiro Takuma, Fumihito Ono, Naomichi Matsumoto, Ryota Hashimoto, Hitoshi Hashimoto, Takanobu Nakazawa

**Affiliations:** 1 Laboratory of Molecular Neuropharmacology, Graduate School of Pharmaceutical Sciences, Osaka University, Osaka, 565-0871 Japan; 2 Osaka Medical and Pharmaceutical University, Research and Development Center, Osaka, 569-8686 Japan; 3 Department of Molecular Pharmacology, Graduate School of Pharmaceutical Sciences, Kyoto University, Kyoto, 606-8501 Japan; 4 Department of Human Genetics, Yokohama City University Graduate School of Medicine, Kanagawa, 236-0004 Japan; 5 Faculty of Nutritional Science, Sagami Women’s University, Kanagawa, 252-0383 Japan; 6 Department of Human Genetics, Research Institute, National Center for Global Health and Medicine, Tokyo, 162-8655 Japan; 7 Department of Pathology of Mental Diseases, National Institute of Mental Health, National Center of Neurology and Psychiatry, Tokyo, 187-8553 Japan; 8 Department of Psychiatry, Graduate School of Medicine, Osaka University, Osaka, 565-0871 Japan; 9 Japan Community Health Care Organization Osaka Hospital, Osaka, 553-0003 Japan; 10 Medical Corporation Foster, Osaka, 531-0075 Japan; 11 Laboratory of Molecular Biology, Department of Bioscience, Graduate School of Life Sciences, Tokyo University of Agriculture, Tokyo, 156-8502 Japan; 12 Interdisciplinary Program for Biomedical Sciences, Institute for Transdisciplinary Graduate Degree Programs, Osaka University, Osaka, 565-0871 Japan; 13 Molecular Research Center for Children’s Mental Development, United Graduate School of Child Development, Osaka University, Kanazawa University, Hamamatsu University School of Medicine, Chiba University, and University of Fukui, Osaka, 565-0871 Japan; 14 Department of Cellular and Molecular Pharmacology, Graduate School of Biomedical and Health Sciences, Hiroshima University, Minami‑ku, Hiroshima 734‑8553 Japan; 15 Graduate School of Agriculture and Life Sciences, The University of Tokyo, Tokyo, 113-8657 Japan; 16 Department of Pharmacology, Graduate School of Dentistry, Osaka University, Osaka, 565-0871 Japan; 17 Department of Physiology, Osaka Medical and Pharmaceutical University, Osaka, 569-8686 Japan; 18 Division of Bioscience, Institute for Datability Science, Osaka University, Osaka, 565-0871 Japan; 19 Transdimensional Life Imaging Division, Institute for Open and Transdisciplinary Research Initiatives, Osaka University, Osaka, 565-0871 Japan; 20 Department of Molecular Pharmaceutical Science, Graduate School of Medicine, Osaka University, Osaka, 565-0871 Japan

**Keywords:** Molecular neuroscience, Stem cells

## Abstract

An increasing body of evidence suggests that impaired synapse development and function are associated with schizophrenia; however, the underlying molecular pathophysiological mechanism of the disease remains largely unclear. We conducted a family-based study combined with molecular and cellular analysis using induced pluripotent stem cell (iPSC) technology. We generated iPSCs from patients with familial schizophrenia, differentiated these cells into neurons, and investigated the molecular and cellular phenotypes of the patient’s neurons. We identified multiple altered synaptic functions, including increased glutamatergic synaptic transmission, higher synaptic density, and altered splicing of dopamine D2 receptor mRNA in iPSC-derived neurons from patients. We also identified patients’ specific genetic mutations using whole-exome sequencing. Our findings support the notion that altered synaptic function may underlie the molecular and cellular pathophysiology of schizophrenia, and that multiple genetic factors cooperatively contribute to the development of schizophrenia.

## Introduction

Schizophrenia is a severe neuropsychiatric disorder characterized by positive symptoms such as hallucinations and delusions, negative symptoms such as decreased motivation and emotional dullness, and cognitive dysfunction. Abnormalities in the formation and function of neural circuits are suggested to be associated with the pathogenesis of this disorder [1–5]; however, the detailed molecular and cellular pathological mechanisms remain largely unclear. Genetic factors may play a major role in the pathogenesis of this disease, and large-scale genome-wide association studies (GWAS) have been conducted so far [6, 7]. Although no specific common single nucleotide polymorphism (SNP) was identified to explain the condition of a large number of patients, these studies have identified common SNPs associated with schizophrenia in genes related to neural function.

In recent years, rare variants, including copy number variants and de novo mutations have been identified to be strongly associated with schizophrenia by large-scale genetic studies [8–11]. In addition to these studies, familial studies may effectively identify disease-associated rare variants transmitted from parents to their offspring [12]. One of the important advantages of familial studies is that, in contrast to studies using unrelated individuals, such as large-scale GWAS, these studies provide sufficient statistical power to detect rare variants even in small sample sizes. Recent family-based studies have identified potential candidate genes for schizophrenia, including several synaptic proteins [12–14].

Induced pluripotent stem cell (iPSC) technology offers an emerging opportunity to study the molecular and cellular mechanisms underlying schizophrenia pathogenesis. iPSC-based studies have recently identified various abnormalities in neuronal development, synaptic development and function, and intracellular signaling pathways [15–20]. Importantly, iPSC technology enables molecular pathological studies of neuronal cells with the same genetic background as the patient. Considering the high heritability factor for familial schizophrenia, modeling familial schizophrenia using iPSC technology can provide an important strategy to unravel the potential causative molecular and cellular pathophysiological mechanisms of the disease. In this study, we generated iPSCs from patients with familial schizophrenia, differentiated these cells into neurons, and investigated the cellular phenotypes of iPSC-derived neurons from patients. We identified multiple altered synaptic functions, including increased frequency and amplitude of miniature excitatory postsynaptic currents (mEPSCs), higher synaptic density, increased AMPA-type glutamate (AMPA) receptor expression, and altered expression ratio of the short and long forms of dopamine D2 receptor (*DRD2*) mRNA in iPSC-derived neurons from patients. We also performed a whole-exome sequencing analysis of the patients’ genomes and identified the patients’ specific genetic mutations. These findings support the notion that altered synaptic functions may underlie the molecular and cellular pathophysiology of schizophrenia, and that multiple genetic factors cooperatively contribute to the development of schizophrenia.

## Materials/subjects and methods

This study was carried out following the Declaration of Helsinki of the World Medical Association and was approved by the Research Ethics Committee of Tokyo University of Agriculture, Osaka University, Osaka Medical and Pharmaceutical University, National Center of Neurology and Psychiatry, the University of Tokyo, and Yokohama City University. All recombinant DNA experiments were reviewed and approved by the Gene Modification Experiments Safety Committee of the respective institutions.

### Subjects

Patients with schizophrenia were recruited at Osaka University Hospital, and diagnosed and assessed by at least two trained psychiatrists according to the Diagnostic and Statistical Manual of Mental Disorders, fourth edition (DSM-IV) criteria based on a structured clinical interview. Symptoms of schizophrenia were assessed using the Positive and Negative Syndrome Scale (PANSS). Written informed consent was obtained from all subjects after the procedures were fully explained.

#### P0482

The proband was an 18-year-old Japanese male patient diagnosed with schizophrenia. The age at onset was 9 years. The patient has 9 years of education and a history of violence. The PANSS scores at the time of sampling were PANSS positive (30); negative (46); general (77). The patient had been treated with antipsychotic medication, but his condition was poorly controlled. The patient was repeatedly admitted and discharged from the hospital.

#### P0481

The patient, the aunt of P0482, was a 49-year-old Japanese female diagnosed with schizophrenia. The age of onset was 38 years old. Impulsive behaviors associated with auditory hallucinations and delusions were sometimes observed. Medication was discontinued at the patient’s discretion.

#### P0480

The patient, the uncle of P0482, was a 45-year-old Japanese male diagnosed with schizophrenia. The age at onset was 34 years.

#### P0479

The patient, the mother of P0482, was a 37-year-old Japanese female diagnosed with schizophrenia. The age at onset was 23 years.

Healthy controls were recruited through local advertisements. Psychiatrically, medically, and neurologically healthy controls were evaluated using the DSM-IV structured clinical interview, non-patient version (SCID-I/NP) that included an assessment of whether the subject had physical problems. Participants were excluded if they had neurological or medical conditions that could potentially affect the central nervous system, such as atypical headache, head trauma with loss of consciousness, epilepsy, seizures, chronic lung disease, kidney disease, chronic hepatic disease, thyroid disease, active stage cancer, or cerebrovascular disease. Neurological and medical conditions were assessed through interviews. C0669 and C0405 were age- and sex-matched controls for P0482 and P0481, respectively.

### Generation of iPSCs

iPSC lines were generated using immortalized B cells obtained from P0482, P0481, C0669, and C0405 as described previously [21, 22]. Plasmid vectors for induction of pluripotency, including 0.63 μg pCE-hOCT3–4, 0.63 μg pCE-hSK, 0.63 μg pCE-hUL, 0.63 μg pCE-mp53DD, and 0.50 μg pCXB-EBNA1 (Addgene, MA, USA) were electroporated in the immortalized B cells using the Nucleofector 2b Device (Lonza, Basel, Switzerland) with the Amaxa Human T-cell Nucleofector Kit (Lonza). Subsequently, the electroporated immortalized B cells were seeded in mitomycin C-treated feeder cells (mouse SL10; Reprocell Inc., Kanagawa, Japan) and cultured for 20−30 days. Colonies of cells similar to human embryonic stem cells were clonally isolated, morphologically selected, subjected to a polymerase chain reaction (PCR)-based analysis of loss of episomal vector, and evaluated for the expression of pluripotent markers using immunocytochemistry (TRA-1–60, TRA-1–81, OCT-4A, and SOX2). As a result, the iPSC clones (clone numbers, C0405-29 (derived from C0405), C0669-4, C0669-12, C0669-15 (derived from C0669), P0481-4, P0481-10 (derived from P0481), P0482-17, and P0482-22 (derived from P0482)) were selected and used for all analyses.

### Neuronal differentiation of iPSCs

The neural induction of iPSC lines into neural stem cells (NSCs) was performed with PSC Neural Induction Medium (Thermo Fisher Scientific, MA, USA), according to the manufacturer’s instructions. For neuronal differentiation, patient- and control-derived NSCs were seeded in Brain Phys Basal Medium (Stemcell Technologies, Vancouver, Canada)-based neuronal differentiation medium containing 1% N2 supplement (Wako, Osaka, Japan), 2% B27 supplement (Thermo Fischer Scientific), 200 μM l-ascorbic acid (Sigma-Aldrich, MO, USA), 1 mM dBcAMP (Wako), 20 ng/mL human brain-derived neurotrophic factor (BDNF; R&D Systems, MN, USA), 20 ng/mL human glial-cell-line-derived neurotrophic factor (GDNF; R&D Systems), 500 ng/mL mouse laminin (Thermo Fisher Scientific), and 1 μM DAPT (Wako) on a 24-well plate coated with 0.1 mg/mL poly-l-ornithine (Sigma-Aldrich), 6.67 μg/mL mouse laminin (Thermo Fisher Scientific), and 6.67 μg/mL human fibronectin (Thermo Fisher Scientific).

### Immunocytochemistry

Immunocytochemistry was performed as described in previous studies [21, 23, 24]. iPSC-derived neurons were cultured for 30−40 days for immunocytochemistry. The analysis of the spine density was performed using data from secondary dendrites that were not overlapped with other dendrites. In our culture conditions, such secondary dendrites were rare, so that we calculated the spine density using all the observed secondary dendrites that were not overlapped with other dendrites. The primary antibodies used for immunocytochemistry were mouse anti-synaptophysin (BD Biosciences, CA, USA, #611880, 1:500), rabbit anti-MAP2 (Merck Millipore, MA, USA, #AB5622, 1:200), rabbit anti-PSD-95 (Cell Signaling Technology, MD, USA, #3450, 1:100), mouse anti-PSD-95 (Merck Millipore, #MABN68, 1:250), mouse anti-TRA-1-60 (Cell Signaling Technology, Stem Light Pluripotency Antibody Kit, #9656 S, 1:1000), mouse anti-TRA-1-81 (Cell Signaling Technology, #9656 S, 1:500), rabbit anti-SOX2 (Cell Signaling Technology, #9656 S, 1:800), and rabbit anti-OCT-4A antibodies (Cell Signaling Technology, #9656 S, 1:800). Hoechst 33258 dye was used to stain nuclei (Calbiochem, CA, USA). Images of the stained cells were acquired using an Olympus FluoView FV1000 confocal microscope (Olympus, Tokyo, Japan) and were analyzed using ImageJ software (NIH, MD, USA) and Adobe Photoshop CS (Adobe Systems, CA, USA).

### Quantitative real-time reverse transcription PCR (RT-PCR)

Total RNA from cultured cells was isolated using the PureLink RNA Mini Kit (Thermo Fisher Scientific) according to the manufacturer’s instructions and was reverse transcribed using Superscript III (Life Technologies, CA, USA). Real-time PCR was performed with SYBR Premix Ex Taq (Takara Bio Inc., Shiga, Japan) using a CFX96 real-time PCR detection system (Bio-Rad Laboratories, CA, USA) as described in a previous study [23]. The expression levels of *MAP2* (forward primer sequence: 5′-CCTGTGTTAAGCGGAAAACC-3′; reverse primer sequence: 5′-AGAGACTTTGTCCTTTGCCTGT-3′), *GRIA1* (forward primer sequence: 5′-GGAAGGACGGGACCAGACAA-3′; reverse primer sequence: 5′-AACGATGCGACCAGACAGGG-3′), *GRIA2* (forward primer sequence: 5′-AGTGCGGAGCCCTCTGTGTT-3′; reverse primer sequence: 5′-ATGGTGTCGCAAGGCTTCCT-3′), *GRIA3* (forward primer sequence: 5′-TGCCAATCTCGCTGCTTTCC-3′; reverse primer sequence: 5′-CGGAGTCCAGGGTCCCATAT-3′), and *GRIA4* (forward primer sequence: 5′-CAGAAGAGCCAGAGGACGGA-3′; reverse primer sequence: 5′-CCTGAGAGGGATCTGGGTGA-3′) were normalized to those of *GAPDH* (forward primer sequence: 5′-CAACGACCACTTTGTCAAGC-3′; reverse primer sequence: 5′-GGTGGTCCAGGGGTCTTACT-3′) and were determined according to the 2^-∆∆Ct^ method.

### Electrophysiology

Whole-cell patch-clamp recordings of iPSC-derived neurons were made as described in previous studies [25, 26]. For measurement of mEPSCs, cells in the culture dish were perfused with an extracellular solution containing 119 mM NaCl, 2.5 mM KCl, 25 mM HEPES, 30 mM d-glucose, 2 mM CaCl_2_, and 2 mM MgCl_2_, pH 7.4. Electrodes were filled with a solution containing 125 mM K-methanesulfonate, 6 mM KCl, 2 mM MgCl_2_, 10 mM HEPES, 0.6 mM EGTA, 3.2 mM Mg-ATP, and 1.2 mM Na-GTP, pH 7.4, and membrane potentials were corrected for the liquid junction potential (9 mV). mEPSCs were recorded from iPSC-derived neurons held at −70 mV in the extracellular solution supplemented with 1 μM TTX (Wako), 1 μM strychnine (Sigma-Aldrich), and 50 μM picrotoxin (RBI, MA, USA). Membrane currents were recorded using MultiClamp 700B Microelectrode Amplifier (Molecular Devices, CA, USA). The records were filtered at 2 kHz and acquired at 10 kHz. The series resistance was monitored during the experiments, and cells were required to have a series resistance of <20 MΩ for inclusion in the analysis. mEPSCs were analyzed using OriginPro 2015 (OriginLab, MA, USA), Clampfit 10.7 (Molecular Devices), and Minianalysis 6.0.3 (Synaptosoft, GA, USA) and were detected by setting the amplitude threshold to 6 pA (root mean square, RMS, noise level <2 pA).

### Western blotting

Western blotting was performed as described in a previous study [23]. Data acquisition and analysis were performed using the LAS4000 image analyzer (GE Healthcare, NJ, USA).

### Whole-exome sequencing

Genomic DNA was extracted from whole blood using a QIAamp DNA Blood Maxi Kit (Qiagen, Hilden, Germany). Whole-exome sequencing was performed as described in a previous study [27]. Genetic variants identified by whole-exome sequencing were confirmed by Sanger sequencing.

### Splicing analysis

The expression levels of *D2S* (forward primer sequence: 5′-GTCTCCTTCTACGTGCCCTT-3′; reverse primer sequence: 5′-GGGCAGCCTCCTTTAGTGGA-3′) and *D2L* (forward primer sequence: 5′-GCAAGCGAGTCAACACCA-3′; reverse primer sequence: 5′-CCTCGGGGTGAGTACAGTTG-3′) were determined by quantitative real-time RT-PCR.

### Statistical analysis

For the statistical analyses for the biochemical and immunocytochemical data, we assessed the assumptions of normality and equality of variance by Shapiro–Wilk test and *F*-test, respectively. We applied Student’s *t*-test for statistical analyses for the biochemical data under the assumptions of normality and equality of variance (Supplementary Table 1). Accordingly, the quantified data from immunohistochemistry, western blotting, and quantitative real-time RT-PCR were statistically analyzed using the Student’s *t-*test. Electrophysiological data were statistically analyzed using the Kolmogorov–Smirnov test. All tests were two-sided, and the significance level was set at *P* < 0.05. Statistical analyses were performed using the StatView software (SAS Institute, NC, USA).

## Results

### Generation of iPSC lines from patients with familial schizophrenia

**Fig. 1:**
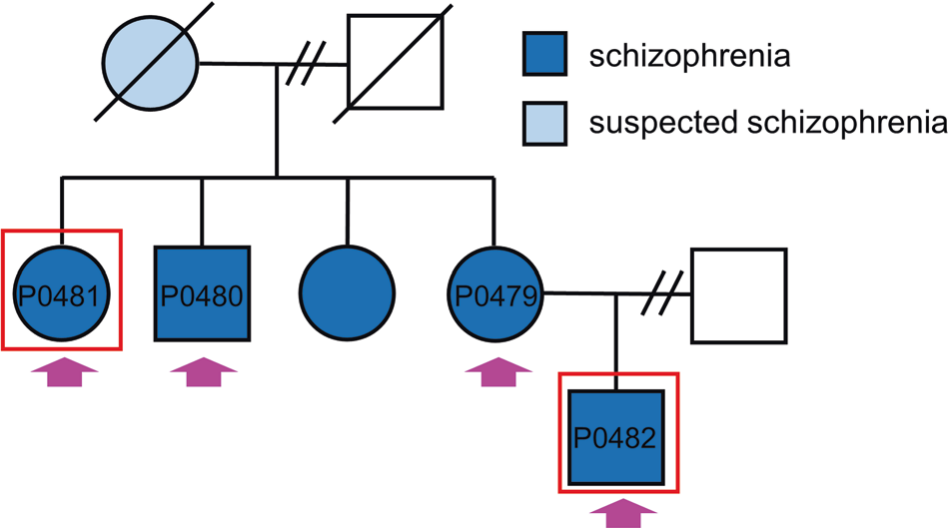
Pedigree of a family with schizophrenia. We performed whole-exome sequencing on four patients (P0479, P0480, P0481, and P0482) indicated by pink arrows. iPSCs were generated from two patients (P0481 and P0482) indicated by red boxes. Blue and light blue represent patients with schizophrenia and suspected schizophrenia, respectively.

We established iPSC lines from patients with familial schizophrenia and psychiatrically healthy age- and sex-matched participants using a non-integrating approach with episomal vectors expressing OCT3/4, SOX2, KLF4, L-MYC, LIN28, a dominant-negative form of TP53, and EBNA1 (Fig. 1) [22]. We clonally isolated and morphologically selected each iPSC line and confirmed the expression of pluripotent markers (TRA-1-60, TRA-1-81, SOX2, and OCT-4A) by immunostaining (Supplementary Fig. 1). Based on these criteria, we selected multiple clones (four clones derived from patients with familial schizophrenia (P0481-4, P0481-10, P0482-17, and P0482-22) and four clones derived from healthy controls (C0405-29, C0669-4, C0669-12, and C0669-15) for further analysis.

### Increased frequency and amplitude of mEPSC in iPSC-derived neurons from patients

**Fig. 2:**
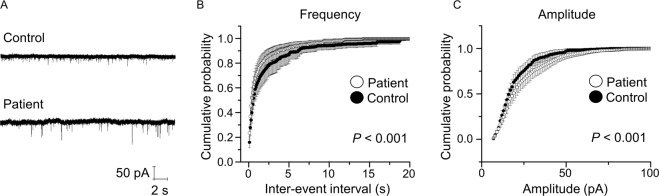
Increased frequency and amplitude of mEPSCs in iPSC-derived neurons from patients. **A** Representative traces of mEPSCs obtained from iPSC-derived neurons from controls and patients. **B** Cumulative probability plot showing a significant shift of the distribution of inter-event interval toward shorter intervals in iPSC-derived neurons from patients. **C** Cumulative probability plot showing a significant shift of the distribution of amplitude toward a larger amplitude in iPSC-derived neurons from patients. *P* < 0.001, Kormogorov–Smirnov test. Data were presented as mean ± standard error of the mean (SEM).

iPSCs were differentiated into NSCs, which were subsequently differentiated into mature neurons. We found no significant differences in the expression of MAP2 between differentiated neurons derived from patients and healthy controls (Supplementary Fig. 2A, B). Abnormal brain functions, such as hallucinations, delusions, decreased motivation, emotional blunting, and cognitive dysfunction in patients with schizophrenia, are believed to be partially caused by impaired synaptic function [3, 28]. We then evaluated excitatory neurotransmission using whole-cell patch-clamp recordings in iPSC-derived neurons from patients and healthy controls, which were cocultured with primary cultured mouse astrocytes to promote neuronal maturation. In the presence of TTX (1 μM), mEPSCs were recorded in iPSC-derived neurons between the period of 28−34 DIV (Fig. 2A) and were blocked by CNQX (10 μM) and AP5 (50 μM) (data not shown). We observed that the frequency of mEPSCs was higher in patient-derived neurons than in healthy control-derived neurons (*P* < 0.001) (Fig. 2B). The amplitude of mEPSCs also increased in patient-derived neurons compared to that in healthy control-derived neurons (*P* < 0.001) (Fig. 2C). These results suggest that, although neural development seems normal, the excitatory synaptic function is impaired in iPSC-derived neurons from patients.

### Higher density of synaptophysin puncta in iPSC-derived neurons from patients

**Fig. 3:**
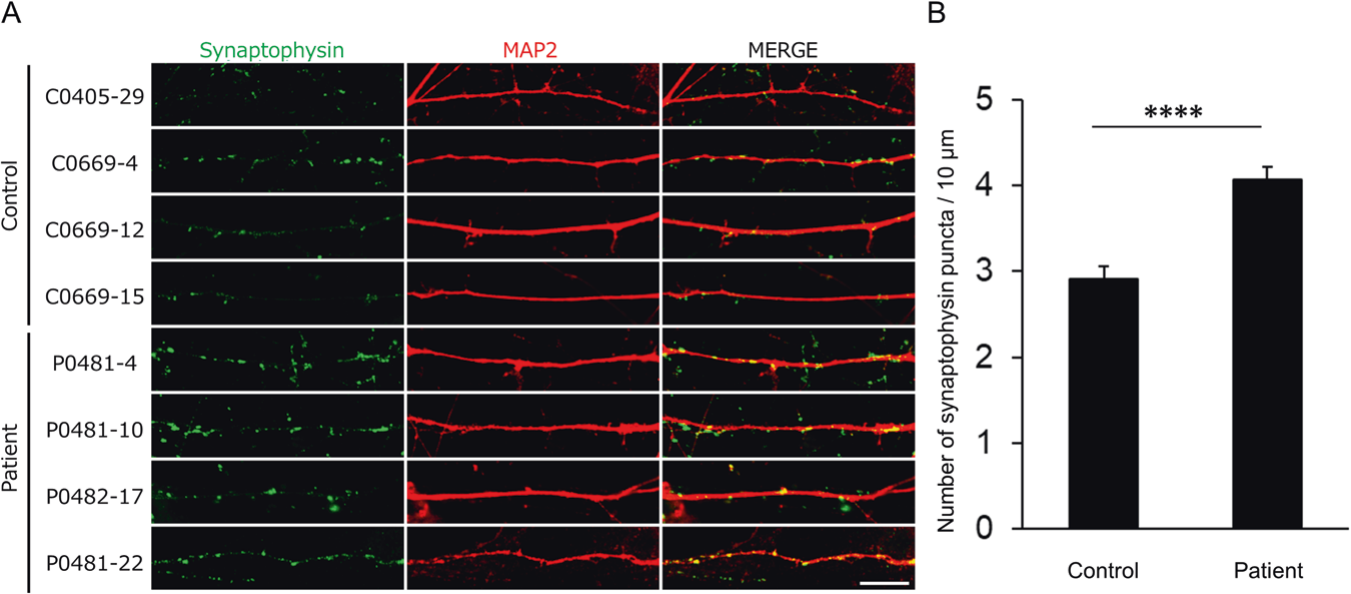
Higher density of synaptophysin puncta in iPSC-derived neurons from patients. iPSC-derived neurons were cultured for 30−40 days for immunocytochemistry. **A** Representative immunostaining images of synaptophysin (green) and MAP2 (red). **B** Quantification of synaptophysin puncta density (control, *n* = 75 dendrites; patients, *n* = 72 dendrites). Scale bar, 10 μm. ****P < 0.0001, Student’s *t*-test. Data were presented as mean ± SEM.

To investigate the localization of presynaptic synaptophysin and postsynaptic PSD-95 in iPSC-derived neurons, we performed immunostaining with antibodies against synaptophysin and PSD-95. We analyzed the density of synaptophysin puncta in iPSC-derived neurons from patients and healthy controls and found that the density of synaptophysin puncta was significantly higher in iPSC-derived neurons from patients than in iPSC-derived neurons from healthy controls (*P* < 0.0001) (Fig. 3A, B). We did not detect postsynaptic PSD-95 puncta in iPSC-derived neurons from patients and healthy controls (data not shown), suggesting that, although mEPSCs were well detected, presynaptic synaptophysin puncta might represent relatively immature synapses. In any case, considering that the frequency of mEPSCs is partially associated with synapse number [29], the increased frequency of mEPSCs in iPSC-derived neurons from patients may be attributable to the higher number of synapses as revealed by the higher density of synaptophysin puncta.

### Higher expression of AMPA receptors in iPSC-derived neurons from patients

**Fig. 4:**
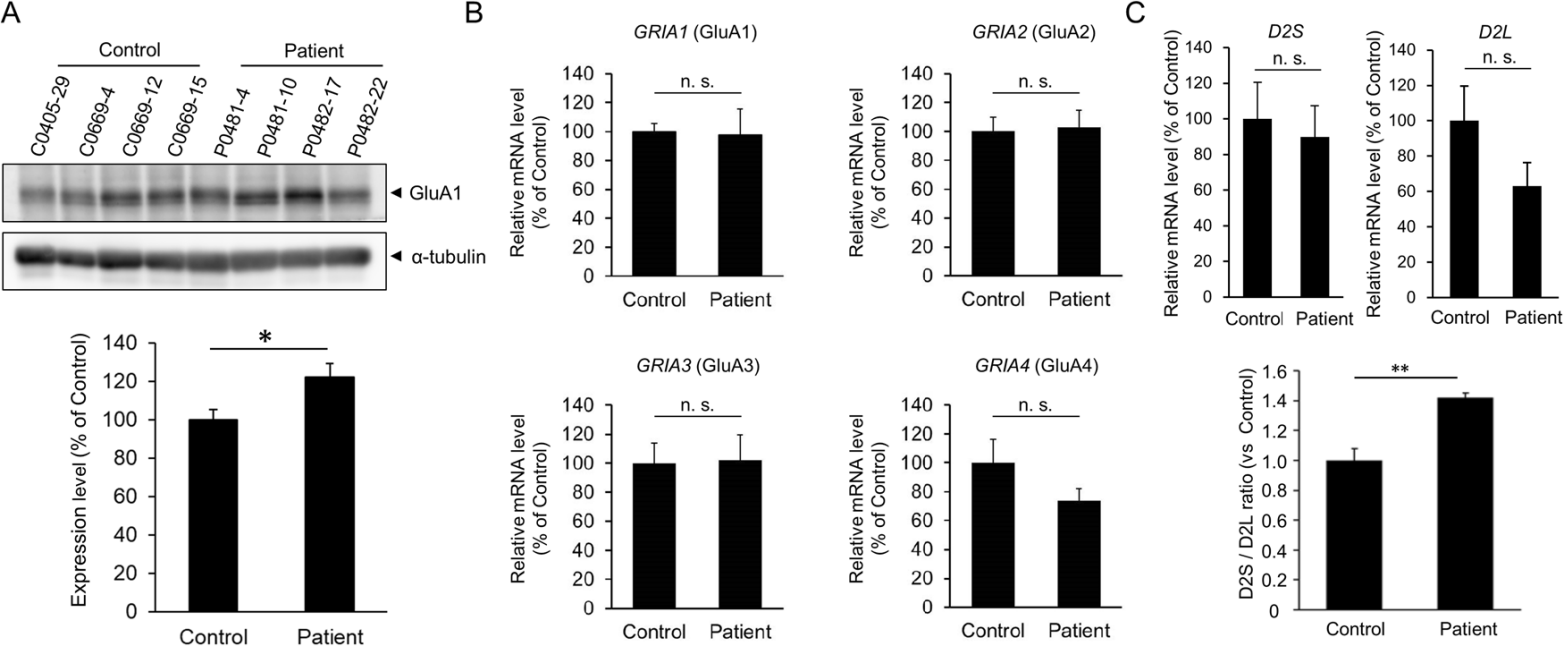
Higher expression of AMPA receptors and impaired splicing of *DRD2* mRNA in iPSC-derived neurons from patients. iPSC-derived neurons were cultured for 30−40 days for the experiments. **A** Protein expression levels of the GluA1 subunit of the AMPA receptor. Representative images of western blotting (upper). Quantification of protein expression levels of GluA1 (lower). Expression levels of GluA1 are normalized to those of α-tubulin (*n* = 4). *P < 0.05, Student’s *t*-test. **B** mRNA expression levels of the subunits of the AMPA receptor. Expression levels were analyzed by quantitative real-time RT-PCR and normalized to those of *GAPDH* (*n* = 4). n. s. not significant, Student’s *t*-test. **C** Increased ratio of the D2S isoform to the D2L isoform in iPSC-derived neurons from patients. Expression levels of D2S and D2L (upper). The ratio of D2S to D2L (lower). The expression levels of D2S and D2L were analyzed by quantitative real-time RT-PCR and normalized to the expression level of *GAPDH* (*n* = 4). **P < 0.01, Student’s *t*-test.

The amplitude of mEPSCs is associated with the expression and activation state of AMPA receptors, which are composed of four subunits, GluA1-4 [30]. We performed western blotting and quantitative real-time RT-PCR to investigate the expression of AMPA receptors. We first analyzed the protein expression of the AMPA receptor and found that the expression of the GluA1 subunit of the AMPA receptor was higher in iPSC-derived neurons from patients than in iPSC-derived neurons from healthy controls (*P* < 0.05) (Fig. 4A). We then examined the expression of mRNA encoding GluA1-4 (gene name: *GRIA1*-*4*) in iPSC-derived neurons from patients and healthy controls using quantitative real-time RT-PCR and found that the mRNA expression level of *GRIA1*-*4* was not significantly different between iPSC-derived neurons from patients and healthy controls (Fig. 4B). These results suggest that the higher protein expression of AMPA receptors may be due to abnormalities in the posttranscriptional regulation of AMPA receptor expression in iPSC-derived neurons from patients.

### Altered splicing of dopamine D2 receptor mRNA (*DRD2*) in iPSC-derived neurons from patients

Genetic factors are believed to be associated with the development of the disease in patients with familial schizophrenia [12]. To examine whether the patients harbored common genetic variants, we conducted whole-exome sequencing of four patients with familial schizophrenia (Fig. 1). We identified the patients’ specific genetic variants at 11 loci that were common to the four patients. These included missense mutations in *TRIT1*, *ATP6V0B*, *FBXO38*, *PCDH9*, *SLC15A1*, *C14orf43*, *WDR93*, *ARMC5*, and *HNRNPM*, deletion of *HSD17B1*, and a nonsense mutation in *ZNF844* (Table 1). Among these mutations, we focus on one of the common patient-specific genetic mutation in *HNRNPM*, whose product, heterogeneous nuclear ribonucleoprotein M (hnRNP M), is involved in the alternative pre-mRNA splicing of *DRD2* [31]. The pre-mRNA of *DRD2* undergoes alternative splicing to generate two isoforms, the short form (D2S) and long form (D2L) [32–35]. We performed a splicing analysis of *DRD2* and found that the expression of D2L tended to be decreased in iPSC-derived neurons from patients, although statistically insignificant (Fig. 4C). The expression of D2S was virtually similar between iPSC-derived neurons from healthy controls and patients (Fig. 4C). Interestingly, the expression ratio of D2S to D2L was significantly higher in iPSC-derived neurons from patients than in iPSC-derived neurons from healthy controls (*P* < 0.01) (Fig. 4C).

**Table 1:** Patients'-specific genetic variants.

Gene	Amino acid change	Chr	Position	Polyphen
*TRIT1*	NM_017646:c.A1034G:p.Y345C	1	40310285	probably damaging 0.999
*ATP6V0B*	NM_004047:c.C5T:p.T2M	1	44440717	probably damaging 0.975
*FBXO38*	NM_030793:c.G153A:p.M51I	5	1.48E + 08	probably damaging 0.979
*PCDH9*	NM_020403:c.C32T:p.A11V	13	67802541	benign 0.208
*SLC15A1*	NM_005073:c.T1115C:p.F372S	13	99360974	probably damaging 1.000
*C14orf43*	NM_001043318:c.C316T:p.R106W	14	74206396	probably damaging 1.000
*WDR93*	NM_020212:c.T1461G:p.C487W	15	90276367	probably damaging 0.999
*ARMC5*	NM_001105247:c.T1493C:p.L498P	16	31475837	benign 0.001
*HSD17B1*	NM_000413:c.873_884del:p.291_295del	17	40706842	deletion
*HNRNPM*	NM_005968:c.G2174A:p.R725Q	19	8553719	probably damaging 0.998
*ZNF844*	NM_001136501:c.T423G:p.Y141X	19	12186358	stop codon

All genetic variants were confirmed by Sanger sequencing.

## Discussion

In this study, we identified abnormal synaptic function and organization, including increased frequency and amplitude of mEPSCs, higher density of synaptic structures, and increased AMPA receptor expression in iPSC-derived neurons from patients. We also observed an altered ratio of D2S to D2L of *DRD2* in iPSC-derived neurons from patients. These results suggest that a combination of synaptic abnormalities may be associated with the pathophysiology of schizophrenia in this family. Importantly, electrophysiological experiments determine the functional impairments in iPSC-derived neurons from patients at the single synapse-level resolution, which would help identify the pathogenesis of schizophrenia at the molecular level.

Excitatory synaptic transmission altered in genetic and proteomic analyses of samples from patients with schizophrenia [36, 37], which may be due to the hypofunction of *N*-methyl-d-aspartate (NMDA) receptors [38]. In contrast to NMDA receptors, the potential role of AMPA receptors in schizophrenia has been controversial [39]. Recent studies have found that the expression of synaptic AMPA receptors is reduced in the auditory cortex of patients with schizophrenia [40, 41] and that mutations in *GRIA2A*, which encodes the GluA2 subunit of the AMPA receptor, are associated with schizophrenia [42]. In contrast to these findings, the mRNA expression of *GRIA2A* and *GRIA4A*, which encode the GluA2 and GluA4 subunits, respectively, is increased in the postmortem brains of patients with schizophrenia [43]. The number of excitatory synapses increases in the caudate nucleus, putamen, and nucleus accumbens in the postmortem brain of patients with schizophrenia [39]. Considering these inconsistent findings, in addition to genetic and postmortem studies, electrophysiological studies of iPSC-derived neurons from patients with schizophrenia are important to elucidate the synaptic pathology in schizophrenia; however, to date, such studies are rare and further research is warranted [15–20]. In this study, in addition to the increased amplitude of mEPSCs (Fig. 2C), we found that the expression levels of AMPA receptors were higher in iPSC-derived neurons from patients than in neurons from healthy controls (Fig. 4A). Although the precise molecular mechanism underlying the increased amplitude of mEPSCs is currently unclear, increased AMPA receptor expression may be related to the increased amplitude of mEPSCs in iPSC-derived neurons from patients. It will be important to examine the synaptic expression level of AMPA receptors and posttranslational modifications of AMPA receptors, including phosphorylation and palmitoylation [44].

The dopamine D2 receptor is considered one of the most relevant gene products and a primary therapeutic target for antipsychotic drugs in schizophrenia. D2L differs from D2S by an additional 29 amino acid insertions within the third cytoplasmic loop of the receptor, which accounts for differential downstream signaling pathways [31]. The postsynaptically localized D2L and presynaptically localized D2S have distinct roles in the regulation of synaptic functions [32–35]. Given that D2S is localized to the presynapse mainly in dopaminergic neurons and regulates dopamine release, a higher expression ratio of D2S to D2L in iPSC-derived neurons from patients may alter the dopaminergic synaptic function. In contrast to D2S, D2L is localized to the postsynapse and regulates the firing properties and intracellular signaling of neurons receiving dopamine input. The cataleptic effects of antipsychotic haloperidol are absent in mice in which the expression of the D2L has been specifically deleted [45], suggesting that D2L is likely targeted by antipsychotics. It may be possible that the uncontrolled symptoms of P0482 by antipsychotic medication might be caused by the decreased D2L expression in iPSC-derived neurons from patients. Interestingly, postmortem brain studies have found that the expression ratio of D2S to D2L in patients with schizophrenia is higher than that in healthy controls [34]. Considering these points, the increased expression ratio of D2S to D2L in iPSC-derived neurons from patients may be the underlying mechanism of the pathophysiology in patients with familial schizophrenia (Fig. 4C). It will be important to generate dopaminergic neurons from patients’ iPSCs and investigate the dopaminergic synaptic function.

The precise molecular mechanism of differential alternative pre-mRNA splicing of *DRD2* in iPSC-derived neurons from patients is currently unclear. We identified a patient-specific mutation in *HNRNPM* (Table 1), whose product regulates alternative pre-mRNA splicing of *DRD2* to generate D2S [31]. The previous study has also found that the generation of D2L is enhanced by Nova-1 and that hnRNP M and Nova-1 are physically interacted with each other to have antagonistic roles in the alternative splicing of DRD2 to generate D2S and D2L [31]. It may be possible that the mutated hnRNP M might interfere with the function of Nova-1 to decrease the expression of D2L in iPSC-derived neurons from patients.

Based on the advantages of familial samples, we identified common patients’ specific genetic variants, which are expected to be associated with the disease with high penetrance. Among the gene loci with these patient-specific mutations identified in this study, the *FBXO38* product is a ubiquitin ligase that is involved in transferring ubiquitin to substrates to control gene expression at the posttranslational level [46]. In iPSC-derived neurons from patients, the function of FBXO38 might have been downregulated by the mutation, resulting in less degradation of the AMPA receptor protein. The underlying mechanism for higher synaptic density in iPSC-derived neurons from patients is also currently unclear. *Pcdh9*-deficient mice show increased dendritic spine number as well as social and recognition deficits [47]. It may be possible that the patient-specific mutation on *PCDH9* might impair the function of PCDH9, resulting in increased dendritic spine number. The functional role of the identified patients’ specific mutations in the altered synaptic function remains largely unclear. It is important to clarify the biological phenotypes of each mutation to clarify the possible relevance of these mutations in the pathophysiology of schizophrenia in this family.

One of the limitations of this study is that there are no available healthy controls in this family; therefore, we cannot determine whether the identified mutations are patients’ specific or not. To circumvent this problem, we investigated the identified patient-specific mutations using the Genome Aggregation Database (gnomAD) and examined whether these mutations have not been previously reported. We found that the genetic variants shown in Table 1 have not been previously reported in the gnomAD. In addition to the genetic variants shown in Table 1, we also identified the genetic variants on *EP400* (132539725, C/T), *ITGA2B* (42461707, G/A), *KIAA0922* (154524568, A/G), *NOC4L* (132632518, C/T), *SNTB1* (121706118, A/G), and *SZT2* (43870202, T/C) in all patients’ samples by whole-exome sequencing. We identified the genetic variants in *EP400* (132539725, C/T, four counts in 191,332 samples), *ITGA2B* (42461707, G/A, four counts in 249,364 samples), *KIAA0922* (154524568, A/G, one count in 251,214 samples), *NOC4L* (132632518, C/T, four counts in 242,848 samples), *SNTB1* (121706118, A/G, one count in 31,396 samples), and *SZT2* (43870202, T/C, two counts in 250,788 samples) in the gnomAD. Although, in this study, we have first focused on the genetic variants shown in Table 1, we assume that the genetic variants in *EP400*, *ITGA2B*, *KIAA0922*, *NOC4L*, *SNTB1*, and *SZT2* might be involved in the pathophysiology of schizophrenia in this family.

In this study, we found experimental evidence suggesting that mutant gene products specific to patients with familial schizophrenia may be involved in the development of the disease by affecting glutamate receptor and dopamine receptor signaling. It is important to clarify the molecular and cellular pathophysiological mechanisms in more detail and to validate the mechanisms using a large sample set. Schizophrenia is a genetically and clinically heterogeneous disease, of which the underlying molecular and cellular pathophysiology is likely to vary from patient to patient [1, 2, 48, 49]. Molecular elucidation of disease-associated rare variants leads to stratification of the disease by molecular pathophysiological mechanisms and ultimately to the development of personalized medicine.

## Supplementary information

Supplementary Table 1

Supplementary information

## References

[1] Birnbaum R, Weinberger DR (2017). Genetic insights into the neurodevelopmental origins of schizophrenia. Nat Rev Neurosci.

[2] Sullivan PF, Daly MJ, O’Donovan M (2012). Genetic architectures of psychiatric disorders: the emerging picture and its implications. Nat Rev Genet.

[3] Forrest MP, Parnell E, Penzes P (2018). Dendritic structural plasticity and neuropsychiatric disease. Nat Rev Neurosci.

[4] Price AJ, Jaffe AE, Weinberger DR (2021). Cortical cellular diversity and development in schizophrenia. Mol psychiatry.

[5] Grant SGN (2019). Synapse diversity and synaptome architecture in human genetic disorders. Hum Mol Genet.

[6] Ripke S, Schizophrenia Working Group of the Psychiatric Genomics Consortium. (2014). Biological insights from 108 schizophrenia-associated genetic loci. Nature.

[7] Ikeda M, Takahashi A, Kamatani Y, Momozawa Y, Saito T, Kondo K (2019). Genome-wide association study detected novel susceptibility genes for schizophrenia and shared trans-populations/diseases genetic effect. Schizophr Bull.

[8] Singh T, and SCHEMA Consortium. Exome sequencing identifies rare coding variants in 10 genes which confer substantial risk for schizophrenia. medRxiv:2020.09.18.20192815 [Preprint]. 2020.

[9] Fromer M, Pocklington AJ, Kavanagh DH, Williams HJ, Dwyer S, Gormley P (2014). De novo mutations in schizophrenia implicate synaptic networks. Nature.

[10] Howrigan DP, Rose SA, Samocha KE, Fromer M, Cerrato F, Chen WJ (2020). Exome sequencing in schizophrenia-affected parent-offspring trios reveals risk conferred by protein-coding de novo mutations. Nat Neurosci.

[11] Kushima I, Aleksic B, Nakatochi M, Shimamura T, Okada T, Uno Y (2018). Comparative analyses of copy-number variation in autism spectrum disorder and schizophrenia reveal etiological overlap and biological insights. Cell Rep..

[12] Glahn DC, Nimgaonkar VL, Raventós H, Contreras J, McIntosh AM, Thomson PA (2019). Rediscovering the value of families for psychiatric genetics research. Mol Psychiatry.

[13] Stertz L, Di Re J, Pei G, Fries GR, Mendez E, Li S (2021). Convergent genomic and pharmacological evidence of PI3K/GSK3 signaling alterations in neurons from schizophrenia patients. Neuropsychopharmacology.

[14] de Vrij FM, Bouwkamp CG, Gunhanlar N, Shpak G, Lendemeijer B, Baghdadi M (2019). Candidate CSPG4 mutations and induced pluripotent stem cell modeling implicate oligodendrocyte progenitor cell dysfunction in familial schizophrenia. Mol Psychiatry.

[15] Balan S, Toyoshima M, Yoshikawa T (2019). Contribution of induced pluripotent stem cell technologies to the understanding of cellular phenotypes in schizophrenia. Neurobiol Dis.

[16] Hoffmann A, Ziller M, Spengler D (2019). Progress in iPSC-based modeling of psychiatric disorders. Int J Mol Sci.

[17] Michael Deans PJ, Brennand KJ (2021). Applying stem cells and CRISPR engineering to uncover the etiology of schizophrenia. Curr Opin Neurobiol.

[18] Nakazawa T, Hashimoto R, Takuma K, Hashimoto H (2019). Modeling of psychiatric disorders using induced pluripotent stem cell-related technologies. J Pharmacol Sci.

[19] Powell SK, O’Shea CP, Shannon SR, Akbarian S, Brennand KJ (2020). Investigation of schizophrenia with human induced pluripotent stem cells. Adv Neurobiol.

[20] Wen Z, Christian KM, Song H, Ming GL (2016). Modeling psychiatric disorders with patient-derived iPSCs. Curr Opin Neurobiol.

[21] Nakazawa T, Kikuchi M, Ishikawa M, Yamamori H, Nagayasu K, Matsumoto T (2017). Differential gene expression profiles in neurons generated from lymphoblastoid B-cell line-derived iPS cells from monozygotic twin cases with treatment-resistant schizophrenia and discordant responses to clozapine. Schizophr Res.

[22] Fujimori K, Tezuka T, Ishiura H, Mitsui J, Doi K, Yoshimura J (2016). Modeling neurological diseases with induced pluripotent cells reprogrammed from immortalized lymphoblastoid cell lines. Mol Brain.

[23] Matsumura K, Seiriki K, Okada S, Nagase M, Ayabe S, Yamada I (2020). Pathogenic POGZ mutation causes impaired cortical development and reversible autism-like phenotypes. Nat Commun.

[24] Nakazawa T, Hashimoto R, Sakoori K, Sugaya Y, Tanimura A, Hashimotodani Y (2016). Emerging roles of ARHGAP33 in intracellular trafficking of TrkB and pathophysiology of neuropsychiatric disorders. Nat Commun.

[25] Kuriu T, Inoue A, Bito H, Sobue K, Okabe S (2006). Differential control of postsynaptic density scaffolds via actin-dependent and -independent mechanisms. J Neurosci.

[26] Okabe S, Kim HD, Miwa A, Kuriu T, Okado H (1999). Continual remodeling of postsynaptic density and its regulation by synaptic activity. Nat Neurosci.

[27] Hashimoto R, Nakazawa T, Tsurusaki Y, Yasuda Y, Nagayasu K, Matsumura K (2016). Whole-exome sequencing and neurite outgrowth analysis in autism spectrum disorder. J Hum Genet.

[28] Obi-Nagata K, Temma Y, Hayashi-Takagi A (2019). Synaptic functions and their disruption in schizophrenia: from clinical evidence to synaptic optogenetics in an animal model. Proc Jpn Acad Ser B, Phys Biol Sci.

[29] Segal M (2010). Dendritic spines, synaptic plasticity and neuronal survival: activity shapes dendritic spines to enhance neuronal viability. Eur J Neurosci.

[30] Malinow R, Malenka RC (2002). AMPA receptor trafficking and synaptic plasticity. Annu Rev Neurosci.

[31] Park E, Iaccarino C, Lee J, Kwon I, Baik SM, Kim M (2011). Regulatory roles of heterogeneous nuclear ribonucleoprotein M and Nova-1 protein in alternative splicing of dopamine D2 receptor pre-mRNA. J Biol Chem.

[32] Radl D, Chiacchiaretta M, Lewis RG, Brami-Cherrier K, Arcuri L, Borrelli E (2018). Differential regulation of striatal motor behavior and related cellular responses by dopamine D2L and D2S isoforms. Proc Natl Acad Sci USA.

[33] Shioda N (2017). Dopamine D2L receptor-interacting proteins regulate dopaminergic signaling. J Pharmacol Sci.

[34] Kaalund SS, Newburn EN, Ye T, Tao R, Li C, Deep-Soboslay A (2014). Contrasting changes in DRD1 and DRD2 splice variant expression in schizophrenia and affective disorders, and associations with SNPs in postmortem brain. Mol Psychiatry.

[35] Khan ZU, Mrzljak L, Gutierrez A, de la Calle A, Goldman-Rakic PS (1998). Prominence of the dopamine D2 short isoform in dopaminergic pathways. Proc Natl Acad Sci USA.

[36] Uno Y, Coyle JT (2019). Glutamate hypothesis in schizophrenia. Psychiatry Clin Neurosci.

[37] McCutcheon RA, Krystal JH, Howes OD (2020). Dopamine and glutamate in schizophrenia: biology, symptoms and treatment. World Psychiatry.

[38] Hardingham GE, Do KQ (2016). Linking early-life NMDAR hypofunction and oxidative stress in schizophrenia pathogenesis. Nat Rev Neurosci.

[39] Roberts RC, McCollum LA, Schoonover KE, Mabry SJ, Roche JK, Lahti AC (2020). Ultrastructural evidence for glutamatergic dysregulation in schizophrenia. Schizophrenia Res.

[40] Beneyto M, Meador-Woodruff JH (2006). Lamina-specific abnormalities of AMPA receptor trafficking and signaling molecule transcripts in the prefrontal cortex in schizophrenia. Synapse.

[41] MacDonald ML, Garver M, Newman J, Sun Z, Kannarkat J, Salisbury R (2020). Synaptic proteome alterations in the primary auditory cortex of individuals with schizophrenia. JAMA Psychiatry.

[42] Gulsuner S, Stein DJ, Susser ES, Sibeko G, Pretorius A, Walsh T (2020). Genetics of schizophrenia in the South African Xhosa. Science.

[43] Dracheva S, McGurk SR, Haroutunian V (2005). mRNA expression of AMPA receptors and AMPA receptor binding proteins in the cerebral cortex of elderly schizophrenics. J Neurosci Res.

[44] Lu W, Roche KW (2012). Posttranslational regulation of AMPA receptor trafficking and function. Curr Opin Neurobiol.

[45] Usiello A, Baik JH, Rougé-Pont F, Picetti R, Dierich A, LeMeur M (2000). Distinct functions of the two isoforms of dopamine D2 receptors. Nature.

[46] Cardozo T, Pagano M (2004). The SCF ubiquitin ligase: insights into a molecular machine. Nat Rev Mol Cell Biol.

[47] Bruining H, Matsui A, Oguro-Ando A, Kahn RS, Van't Spijker HM, Akkermans G (2015). Genetic mapping in mice reveals the involvement of Pcdh9 in long-term social and object recognition and sensorimotor development. Biol Psychiatry.

[48] Howell KR, Law AJ (2020). Neurodevelopmental concepts of schizophrenia in the genome-wide association era: AKT/mTOR signaling as a pathological mediator of genetic and environmental programming during development. Schizophr Res.

[49] Hiroi N, Yamauchi T (2019). Modeling and predicting developmental trajectories of neuropsychiatric dimensions associated with copy number variations. Int J Neuropsychopharmacol.

