# Supplementary material for: Multiple alterations in glutamatergic transmission and dopamine D2 receptor splicing in induced pluripotent stem cell-derived neurons from patients with familial schizophrenia
Source: Transl Psychiatry. 2021 Oct 25;11:548. doi: 10.1038/s41398-021-01676-1 (PMC8547217; doi:10.1038/s41398-021-01676-1)
Supplement: Supplementary file 2 — Supplementary information [file 41398_2021_1676_MOESM2_ESM.pdf]

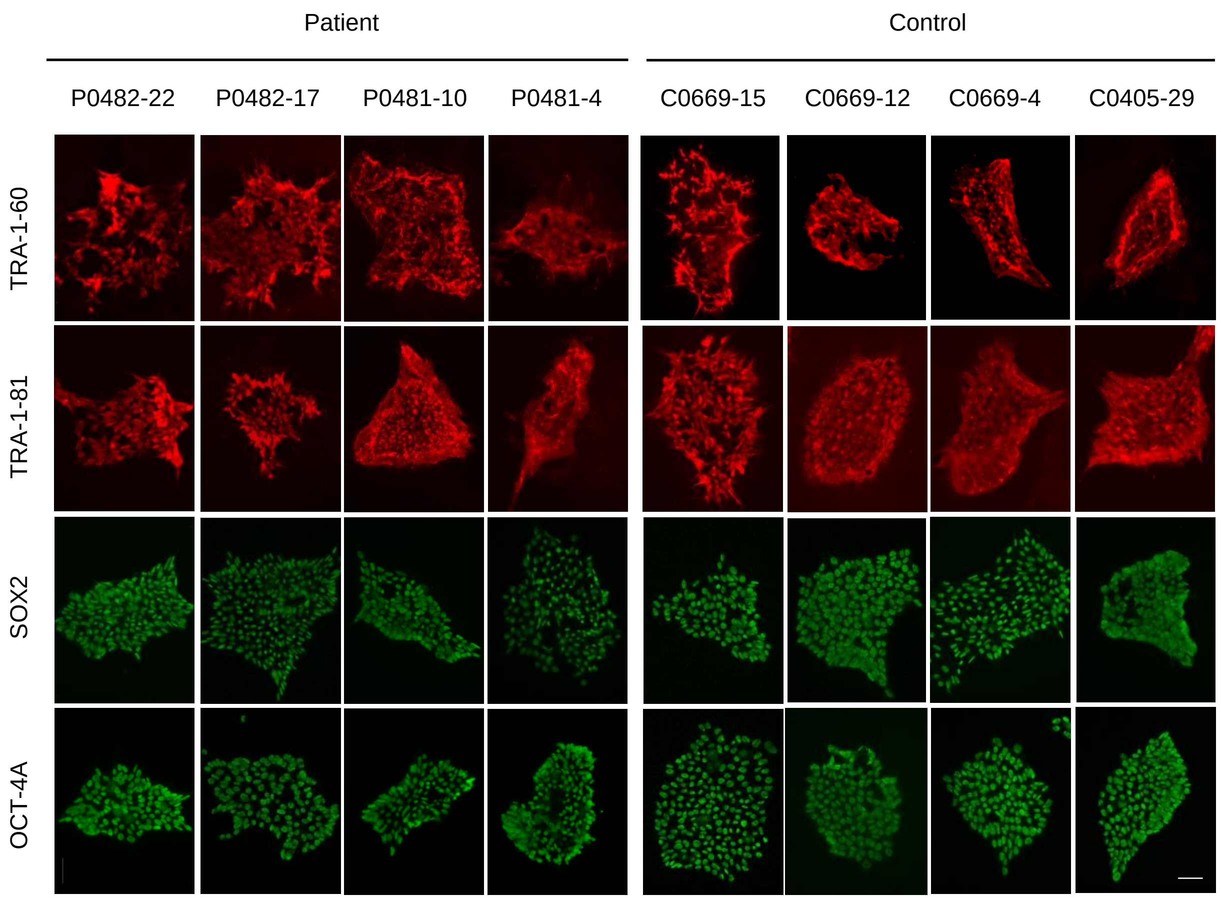

Supplementary Fig. 1 Representative immunostaining images of iPSCs from patients and healthy controls used in this study. iPSCs were stained for pluripotent markers (TRA-1-60, TRA-1-81, SOX2, and OCT-4A). Scale bar, 50 μm.

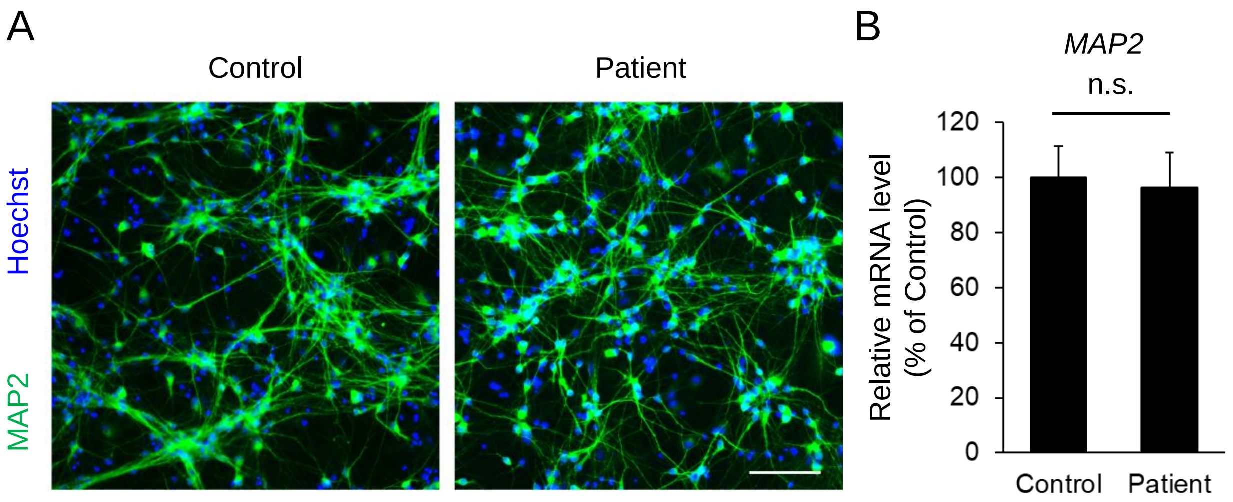

Supplementary Fig. 2 Neural differentiation of iPSC derived from patients and healthy controls. **A** Representative images of differentiated neurons. Cells were stained with a neuronal marker, MAP2. Nuclei were stained with Hoechst 33258. Scale bar, 100 μm. **B** Quantification of the mRNA expression of *MAP2* in differentiated neurons. The expression levels of *MAP2* were analyzed by real-time RT-PCR and normalized to those of *GAPDH* (each n = 4 lines). n.s., not significant, Student's *t*-test.
